# The cold‐water immersion recovery–adaptation paradox: Reconciling acute parasympathetic and analgesic benefits with chronic hypertrophy attenuation

**DOI:** 10.1113/EP094042

**Published:** 2026-08-29

**Authors:** Jose Francisco Tornero‐Aguilera, Jose Lozano‐Meca, Miguel López‐Moreno, Edgar Simón Sancho‐Haro, Mario Muñoz‐López, Eneko Baz‐Valle, José Francisco López‐Gil, Rodrigo Yáñez‐Sepúlveda, Vicente Javier Clemente‐Suárez

**Affiliations:** ^1^ Department of Sport Sciences Faculty of Sport and Health Sciences Fit Generation Research Institute Andorra la Vella Andorra; ^2^ Department of Nursing and Physiotherapy Faculty of Nursing and Physiotherapy University of the Balearic Islands Palma de Mallorca Spain; ^3^ Department of Nutrition and Dietetics Faculty of Sport and Health Sciences Fit Generation Research Institute Andorra la Vella Andorra; ^4^ Faculty of Education and Social Sciences University Andrés Bello Viña del Mar Chile; ^5^ School of Medicine University Espíritu Santo Samborondón Ecuador; ^6^ Faculty of Health Sciences Universidad Autónoma de Chile Temuco Chile

**Keywords:** cold‐water immersion, heart rate variability, mTORC1 signalling, muscle hypertrophy, resistance training

## Abstract

Cold‐water immersion (CWI) is among the most widely used post‐exercise recovery modalities in elite sport and clinical rehabilitation, yet a fundamental paradox now emerges from the evidence: the same protocols that accelerate parasympathetic reactivation and reduce perceived muscle soreness also suppress the molecular and cellular machinery of skeletal‐muscle hypertrophy when applied repeatedly across resistance‐training mesocycles. This narrative review synthesises the multi‐axis acute physiological response to CWI, the meta‐analytic evidence supporting its acute recovery benefits, and the molecular, cellular and longitudinal evidence documenting its chronic attenuation of resistance‐training adaptations. Acute CWI (10–15°C, 10–15 min) consistently augments parasympathetic reactivation, reduces soreness within 24–72 h (network meta‐analytic SUCRA 88%) and modulates inflammatory time‐courses. In parallel, equivalent protocols attenuate post‐resistance‐exercise mechanistic target of rapamycin complex 1 signalling, ribosomal biogenesis and satellite‐cell‐mediated myonuclear addition, translating into reduced type II fibre cross‐sectional area and modest impairments of strength gain (effect size −0.23). Endurance‐related microvascular and mitochondrial adaptations appear less affected and may be selectively enhanced. A protocol‐by‐goal decision framework links CWI parameters – water temperature, duration, body coverage and temporal placement – to expected adaptive outcomes across hypertrophy, strength, endurance and concurrent training contexts. CWI is neither uniformly beneficial nor uniformly harmful; its effects are predictable and depend on the training goal. CWI should be used when fast recovery between sessions matters most, and withheld after resistance training when muscle growth is the main goal of the training block.

## Abstract (highlights)

- **What is the topic of this review?**
  This review asks how the divergent acute (parasympathetic and analgesic) and chronic (anti‐anabolic) effects of post‐exercise cold‐water immersion can be reconciled into a single mechanistically grounded prescription framework.
- **What advances does it highlight?**
  Acute cold‐water immersion (10–15°C, 10–15 min) reliably augments parasympathetic reactivation and reduces perceived soreness, while the same protocols, applied repeatedly after resistance training, attenuate mTORC1 signalling and type II fibre hypertrophy. The protocol‐by‐goal framework presented here translates these mechanistic findings into context‐specific, evidence‐based clinical and applied prescriptions.

## 1 INTRODUCTION

The recovery industry – both clinical and consumer – has converged on cold‐water immersion (CWI) as one of the most visible and commercially proliferated interventions for accelerating return‐to‐performance after exercise (Veen et al., 2026). Cold‐plunge tubs are now standard fixtures in professional sport facilities across European football, rugby and athletics; they appear in commercial wellness chains in urban centres; and an estimated several million CWI sessions are performed weekly in healthy adult populations across Europe, North America and Oceania (Allan et al., 2022). The expansion of recreational CWI has been propelled by social‐media‐driven wellness culture, the popularisation of Wim Hof‐style cold‐exposure protocols and an increasingly vocal consumer discourse that has conflated therapeutic cryotherapy, athletic recovery and general health promotion into a single undifferentiated endorsement of cold (Almahayni & Hammond, 2024; Versey et al., 2013; Wakabayashi et al., 2025). Behind all these uses sits a long‐standing assumption that has rarely been subjected to systematic scrutiny within a single framework: that the modality, by attenuating inflammation and perceived muscle soreness, must necessarily benefit subsequent training quality, adaptation and long‐term performance. As this review will show, that assumption holds for acute recovery but breaks down for chronic adaptation: applied repeatedly after resistance training, CWI can blunt the very muscle growth that the training is designed to stimulate.

The evidence assembled over the past decade complicates this assumption as a generalisation while preserving it as a context‐specific truth. Three convergent lines sharpen the picture. First, meta‐analytic evidence confirms that acute post‐exercise CWI (10–15°C, 10–15 min) accelerates parasympathetic reactivation on time‐ and frequency‐domain heart rate variability (HRV) indices, reduces delayed onset muscle soreness (DOMS) within 24–72 h, and modulates inflammatory time courses favourably for next‐session readiness (Cain et al., 2025; Chen et al., 2024; Galvez‐Rodriguez et al., 2025; Moore et al., 2022). Second, mechanistic and longitudinal evidence shows that comparable protocols, applied repeatedly after resistance training across mesocycles, attenuate mechanistic target of rapamycin complex 1 (mTORC1) signalling, reduce ribosomal biogenesis, impair satellite‐cell‐mediated myonuclear addition and reduce type II fibre cross‐sectional area gains (Fyfe et al., 2019; Grgic, 2023; Piñero et al., 2024; Roberts et al., 2015). Third, a 2024 International Olympic Committee critical review concluded that direct human evidence for cryotherapy as a regenerative intervention for soft‐tissue injury is essentially absent, with mechanistic claims resting largely on animal extrapolation (Racinais et al., 2024).

Together these findings produce what this review terms the recovery–adaptation paradox: a single intervention improves the acute markers practitioners use to judge recovery – soreness, autonomic balance, perceived freshness, creatine kinase normalisation – while suppressing the molecular and cellular substrates of chronic skeletal‐muscle adaptation (Fyfe et al., 2019; Piñero et al., 2024; Rantala & Chaillou, 2019). This is not a methodological artefact but an emergent property of the heterogeneous responses CWI elicits across vascular, neural, inflammatory, anabolic and perceptual domains, each operating on a distinct time scale (Ihsan et al., 2020; Rousse et al., 2025). The acute benefits are concentrated in the first 24–72 h and are largely reversible; the chronic costs accumulate insidiously across weeks of systematic post‐resistance CWI and become difficult to recover within the closed mesocycle (Choo et al., 2022; Malta et al., 2021; Piñero et al., 2024).

The mTORC1 pathway integrates mechanical, nutritional and hormonal signals to regulate muscle protein synthesis after resistance exercise (detailed in section 5). Cooling skeletal muscle by 2–5°C after resistance exercise slows enzyme kinetics, reduces mTORC1 activation and attenuates the translational machinery needed to capitalise on the post‐exercise muscle protein synthesis signal (Fyfe et al., 2019; Petersen & Fyfe, 2021; Rantala & Chaillou, 2019; Roberts et al., 2015).

This review addresses three questions the field has raised but rarely synthesised within one practitioner‐accessible framework. First, where does the paradox arise mechanistically – which molecular and cellular pathways are simultaneously accelerated and suppressed by post‐exercise CWI? Second, under what protocol conditions (temperature, duration, body coverage, timing) does each side dominate? Third, how can these signals be assembled into an evidence‐based decision framework that lets clinicians, exercise physiologists and coaches deploy CWI with intention rather than habit? The scope is bounded to post‐exercise CWI in healthy adults, with particular attention to resistance‐ and concurrent‐training contexts; thermoregulatory, therapeutic and recreational cold exposure are addressed only where directly relevant.

Throughout this review, CWI denotes whole‐ or partial‐body submersion in water at 5–15°C for 5–20 min applied within minutes to hours after exercise. This excludes whole‐body cryotherapy chambers (cold air, −100°C to −140°C, with a substantially different intramuscular cooling profile), localised ice‐pack application (Bleakley et al., 2004) and cold‐water exercise. Acute effects are those occurring within hours to ∼72 h of a single exposure; chronic effects are adaptation phenotypes (hypertrophy, strength, ${\dot{V}}_{O_{2}\max}$, fibre type) following repeated exposures across multi‐week training cycles (Machado et al., 2016; Yamane et al., 2015).

## 2 METHODS

This narrative review was conducted in accordance with established best practices for synthesis in exercise physiology. Electronic database searches were performed in PubMed/MEDLINE, Web of Science Core Collection and Cochrane CENTRAL from January 2010 to April 2026, with targeted extensions to 2000 for foundational molecular biology and thermoregulation studies. The 2010 start date reflects the fact that the modern CWI evidence base – particularly the molecular signalling and longitudinal resistance‐training studies that define the recovery–adaptation paradox – emerged almost entirely from 2010 onward. The comparatively few earlier foundational papers on muscle protein‐synthesis signalling, thermoregulation and post‐exercise parasympathetic reactivation were captured through the targeted pre‐2010 extensions and through reference mining, so no substantive body of 2000–2009 CWI‐adaptation literature was excluded. The primary search strategy combined MeSH terms and free‐text keywords across four conceptual domains: (i) the intervention (CWI, ice bath, water immersion, cryotherapy, cold therapy, water temperature, cold exposure); (ii) the acute outcome axis (HRV, RMSSD, autonomic recovery, delayed onset muscle soreness, DOMS, creatine kinase, perceived exertion, inflammation, interleukin‐6 (IL‐6), tumour necrosis factor‐α, C‐reactive protein); (iii) the chronic adaptation axis (mTORC1, mTOR signalling, muscle protein synthesis, hypertrophy, muscle fibre cross‐sectional area, strength, satellite cells, ribosomal biogenesis, p70S6K1, eukaryotic translation initiation factor 4E‐binding protein 1 (4E‐BP1)); and (iv) training context (resistance training, endurance training, concurrent training, sport recovery, in‐season).

A representative PubMed string combined the four domains – for example, (‘cold water immersion’ OR ‘ice bath’ OR cryotherapy) AND (‘mTOR’ OR ‘muscle protein synthesis’ OR hypertrophy OR ‘HRV’ OR DOMS OR recovery) AND (‘resistance training’ OR ‘strength training’ OR exercise OR sport). Reference lists of identified systematic reviews, network meta‐analyses and key narrative reviews were screened for additional primary and mechanistic studies. Evidence was weighted hierarchically – meta‐analyses of randomised controlled trials (RCTs) highest, then individual RCTs, longitudinal controlled trials, mechanistic human biopsy studies, and finally in vitro or animal data – with preclinical‐only mechanistic claims explicitly flagged and treated with epistemic caution. Studies beyond healthy adults were included only for mechanistic context.

## 3 THE ACUTE PHYSIOLOGICAL RESPONSE TO CWI: A MULTI‐AXIS CASCADE

**FIGURE 1:**
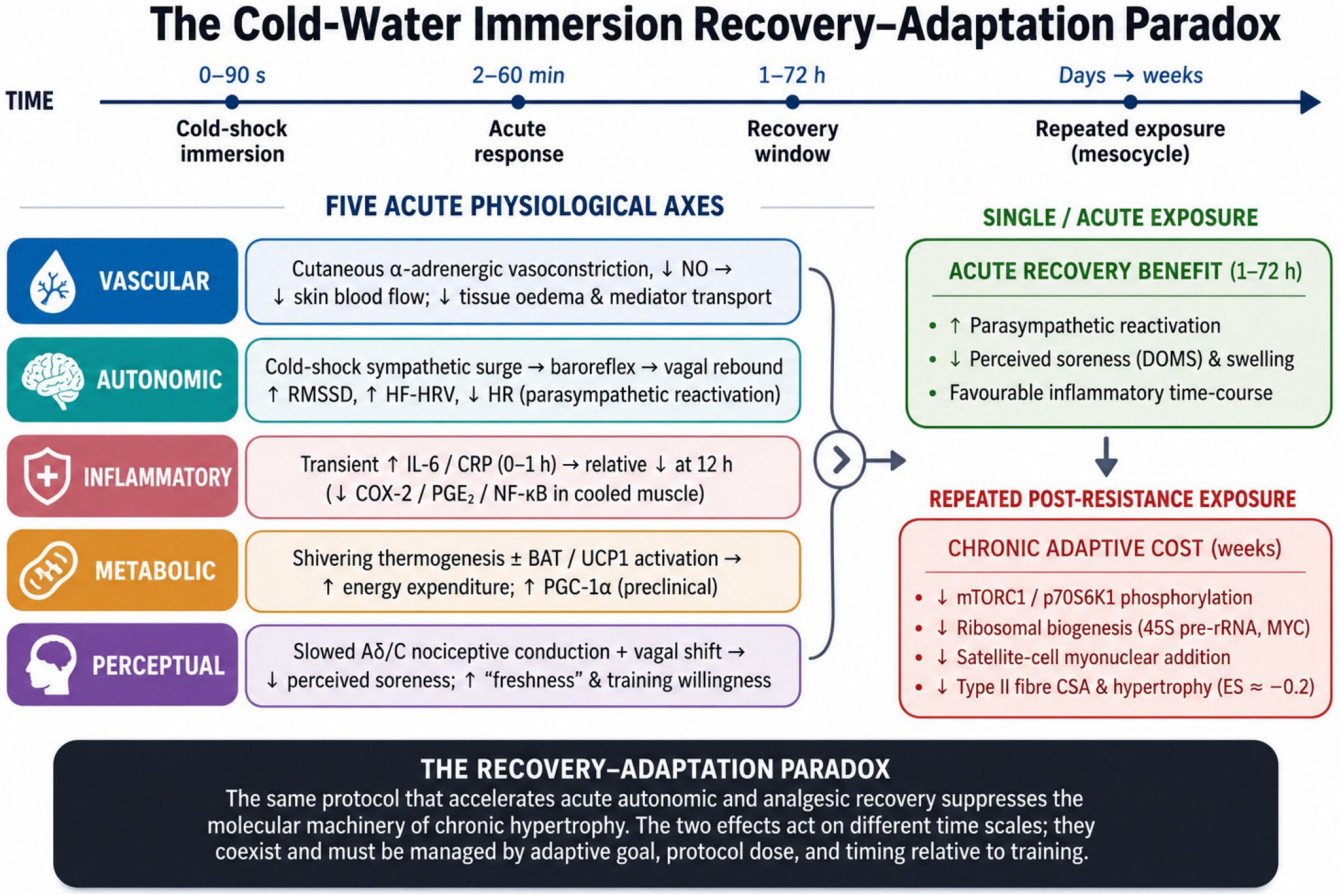
The cold‐water immersion recovery–adaptation paradox: integration of the five acute physiological axes (vascular, autonomic, inflammatory, metabolic, perceptual) along the immersion‐to‐mesocycle time course. Acute, largely reversible benefits dominate the first 1–72 h after a single exposure; repeated post‐resistance‐exercise exposure across weeks drives the chronic attenuation of mTORC1‐dependent hypertrophy that defines the paradox.

Although the five physiological responses to CWI are presented below under separate headings for clarity, they do not occur in isolation. They are activated in an overlapping, time‐ordered cascade that begins within seconds of immersion and, when the exposure is repeated across a training block, ultimately shapes chronic adaptation. Figure 1 integrates these axes along a single time axis – from the cold‐shock response (0–90 s), through the acute autonomic, vascular, inflammatory, metabolic and perceptual responses (minutes to ∼72 h), to the cumulative effect of repeated post‐resistance‐exercise exposure across weeks – and shows how the same cascade that produces the acute recovery benefit also drives the chronic adaptive cost that defines the paradox. The subsections that follow detail each axis in turn but should be read as components of this single integrated time course rather than as independent phenomena.

### 3.1 The vascular axis: Cutaneous vasoconstriction, oedema reduction and microvascular dynamics

Within 20–30 s of immersion in water at 10–15°C, intense cutaneous vasoconstriction is initiated through α‐adrenergic activation and simultaneous inhibition of the nitric oxide (NO) vasodilatory pathway (Rowell, 1974; Wakabayashi et al., 2025; Wilcock et al., 2006). Cutaneous (skin) blood flow falls sharply in the distal limbs within the first minute and remains substantially below resting values for the full duration of the exposure; in the context of cooling, the reduction in peripheral perfusion is driven predominantly by this fall in skin blood flow rather than by an equivalent reduction in muscle blood flow, although the steep thermal gradient it establishes does secondarily lower intramuscular perfusion and metabolic rate as cooling progresses. Reduced peripheral perfusion has two immediate physiological consequences relevant to athletic recovery. First, it creates a steep transmembrane temperature gradient that propagates inward from the subcutaneous compartment to the skeletal‐muscle belly over approximately 20–30 min, cooling intramuscular temperature by 2–5°C depending on submersion depth, adipose thickness and water temperature (Machado et al., 2016; Roberts et al., 2015; Vieira et al., 2016). Second, it mechanically compresses the oedematous fluid accumulating in the interstitial space of recently exercised muscle by reducing capillary hydrostatic pressure, attenuating the net filtration pressure driving fluid from the vascular to the interstitial compartment, and – when the immersion includes hydrostatic compression above knee level – assisting lymphatic drainage through external pressure gradients (Schaser et al., 2007; Wilcock et al., 2006). This oedema‐reduction mechanism is a major contributor to the perceived analgesic benefit of CWI in the 24–48 h post‐exercise window, where swelling‐associated mechanoreceptor activation contributes substantially to perceived soreness (Bleakley et al., 2012).

At the microvascular level, an important distinction exists between the acute response – which suppresses peripheral perfusion – and the adaptive response to repeated CWI during sustained training. A within‐subject trial by Ihsan et al. (2020) demonstrated that the leg receiving 15 min of 10°C CWI after each of 3‐weekly endurance sessions over 4 weeks exhibited significantly higher microvascular reperfusion rates (measured by contrast‐enhanced ultrasound) than the untreated contralateral control leg (Ihsan et al., 2016, 2020). This finding of enhanced rather than attenuated microvascular adaptation with repeated CWI during endurance training is mechanistically plausible through a hormetic cold‐stress mechanism: repeated acute reductions in perfusion followed by reactive hyperaemia may represent a training‐like stimulus for capillary remodelling analogous to the metabolic perturbation that drives angiogenesis during endurance training. This distinction between acute (suppressive) and chronic (potentially adaptive) microvascular effects is one of the key factors underpinning the differential sensitivity of endurance versus resistance adaptations to CWI, discussed in section 6.

### 3.2 The autonomic axis: From cold shock to parasympathetic dominance

The autonomic response to CWI is characteristically biphasic and constitutes the most clinically relevant and best‐characterised benefit of the modality for athletic recovery. The initial cold‐shock response – involuntary gasping, hyperventilation, tachycardia and a transient surge in circulating catecholamines – is mediated by cutaneous thermoreceptor (TRPM8, TRPA1) activation and sympatho‐adrenal stimulation occurring within the first 30–90 s of immersion (Flouris & Schlader, 2015; Wakabayashi et al., 2025). This response accounts for the primary cardiovascular safety risk of CWI: the sympathetic surge combined with peripheral vasoconstriction elevates systemic vascular resistance and cardiac afterload transiently, and in susceptible individuals may provoke arrhythmia or sudden cardiac death, particularly in outdoor cold‐water environments where drowning risk compounds cardiac risk (Faivre‐Rampant et al., 2024). In supervised laboratory settings with healthy trained adults, this initial sympathetic period is brief and self‐limiting. Beyond thermoreception, cold immersion also directly stimulates cutaneous and deep nociceptors in addition to TRPM8/TRPA1 thermoreceptors, and this nociceptive afferent barrage contributes to the initial sympatho‐excitatory cold‐shock response and, through spinal and supraspinal reflex pathways, to the subsequent autonomic adjustments – a nociceptive component that is frequently overlooked when the autonomic response to CWI is attributed to thermoreception and baroreflex activation alone (Cheung et al., 2003; Flouris & Schlader, 2015).

Within 2–3 min of stable immersion, the cold‐shock response gives way to a profound parasympathetic dominance mediated by baroreflex activation. Cold‐induced cutaneous vasoconstriction increases central blood volume and mean arterial pressure, stimulating arterial baroreceptors that reflexly augment vagal outflow to the sinoatrial node (Buchheit et al., 2009; Rowell, 1974). This produces rapid reductions in heart rate and measurable increases in HRV indices reflecting cardiac parasympathetic modulation: the root mean square of successive R–R interval differences (RMSSD) rises substantially, high‐frequency spectral power (0.15–0.40 Hz) increases, and the low frequency/high frequency (LF/HF) ratio normalises toward parasympathetic dominance (Buchheit et al., 2009; Galvez‐Rodriguez et al., 2025; Stanley et al., 2012). The Galvez‐Rodriguez et al. (2025) systematic review, synthesising 12 randomised trials, confirmed that all 12 included studies reported parasympathetic reactivation following post‐exercise CWI, with six demonstrating statistically significant differences versus passive recovery on at least one HRV index (all *P* < 0.05) and eight reporting moderate‐to‐large standardised effect sizes. The effect persists for 30–60 min after emergence from immersion, providing a window in which subjective recovery and training willingness are reliably enhanced. This parasympathetic window has real operational significance in the multi‐session day scenario: an athlete completing morning and afternoon training sessions can use CWI between sessions to partially restore the autonomic modulation that underpins high‐quality afternoon performance.

### 3.3 The inflammatory axis: A nuanced bi‐directional time course

The inflammatory response to acute CWI is bi‐directional and time‐dependent, resolving apparent contradictions in the older literature. The 2025 meta‐analysis by Cain et al. (2025), pooling 11 RCTs in healthy adults, documented significantly increased circulating inflammatory markers (IL‐6, C‐reactive protein, white cell count) at the immediate and 1‐h time points (standardised mean differences 1.0–1.3; *P* < 0.01), consistent with a cold‐stress transient, followed by relative suppression of inflammatory burden at 12 h versus passive recovery (Cain et al., 2025; Gleeson et al., 2011; Peake et al., 2017). Mechanistically, CWI introduces a brief, self‐limiting cold‐stress inflammatory signal, after which reduced activity of temperature‐sensitive enzymes including cyclooxygenase‐2 (COX2) and reduced prostaglandin E2 (PGE2) and nuclear factor kappa‐B (NF‐κB) activation in cooled muscle produce a relative suppression that is smaller and shorter‐lived than the exercise‐induced response it overlaps (Gleeson et al., 2011; Rousse et al., 2025).

This pattern has a critical implication for the paradox. The same NF‐κB and prostaglandin cascade that CWI suppresses in the 12‐h window is not merely an inflammatory nuisance: PGE2 and IL‐6 released from mechanically stimulated fibres are upstream activators of the satellite‐cell cascade required for myonuclear addition and true hypertrophy (Chargé & Rudnicki, 2004; Serrano et al., 2008). Suppressing this cascade accelerates resolution of soreness but simultaneously delays or attenuates the anabolic phase of muscle remodelling (Fyfe et al., 2019; Piñero et al., 2024). This coupling of anti‐inflammatory benefit to anti‐anabolic cost is the deepest explanation of the paradox and is directly analogous to the trade‐off of chronic NSAID use during resistance training (Gleeson et al., 2011; Trappe et al., 2011).

### 3.4 The metabolic axis: Thermogenesis, brown adipose tissue and energy expenditure

Acute CWI raises metabolic rate mainly through shivering thermogenesis and, in cold‐acclimated individuals with brown adipose tissue (BAT), through UCP1‐mediated non‐shivering thermogenesis; the energy cost of a single session (∼50–100 kcal) is negligible for performance or body composition. Repeated cold may upregulate BAT and skeletal‐muscle peroxisome proliferator‐activated receptor γ coactivator 1‐α (PGC‐1α) in animal models, but translation to athletes – who generally have low BAT activity – remains poorly characterised (Cannon & Nedergaard, 2004; Puigserver & Spiegelman, 2003).

### 3.5 The perceptual axis: Freshness, pain modulation and decision behaviour

A fifth physiological axis – the perceptual – warrants explicit placement alongside the four canonical responses because it mediates the most immediate and practitioner‐visible outcome of CWI: the subjective sense of restored readiness. The cold‐shock response, the rapid reduction in skin and muscle temperature, the analgesic effect of slowed nociceptive conduction velocity in Aδ and C fibres, and the subsequent parasympathetic shift collectively produce a distinctive subjective state recognisable to practitioners as ‘freshness’ or ‘reset’ (Cheung et al., 2003; Flouris & Schlader, 2015). The perceptual axis is functionally important not just as an outcome but as a mediator of training behaviour: athletes who perceive themselves to have recovered are more willing to commit to high‐intensity subsequent sessions, to undertake additional training volume and to accept coaching‐imposed performance demands (Chen et al., 2024; Moore et al., 2022). Whether this perceptual benefit translates into objective adaptation gains – by enabling additional high‐quality training – or instead drives maladaptive over‐training by masking the actual tissue‐level state of recovery, is a context‐dependent question that the decision framework in section 7 addresses explicitly. The five acute physiological axes and their integration across the immersion‐to‐mesocycle time course are summarised in Figure 1.

## 4 THE ACUTE RECOVERY SIDE OF THE PARADOX: EVIDENCE AND MECHANISMS

### 4.1 Parasympathetic reactivation and HRV

HRV is the most widely used non‐invasive marker of autonomic recovery status in elite sport, and the evidence linking post‐exercise CWI to parasympathetic reactivation is the most methodologically robust in the CWI literature. The dominant time‐domain metric in CWI studies is RMSSD – the root mean square of successive R–R interval differences – which specifically reflects beat‐to‐beat vagal (parasympathetic) modulation of the sinoatrial node and is now the standard metric in HRV‐guided training studies (Galvez‐Rodriguez et al., 2025; Kiviniemi et al., 2007).

The Galvez‐Rodriguez et al. (2025) systematic review and meta‐analysis, encompassing 12 randomised trials in athletic and recreationally active populations, is the most comprehensive synthesis to date. All 12 included studies reported at least directional parasympathetic reactivation following post‐exercise CWI relative to passive recovery or to pre‐exercise baseline. Six trials reported statistically significant differences (*P* < 0.05) between CWI and passive recovery on RMSSD, high‐frequency power or LF/HF ratio, and eight reported moderate‐to‐large, standardised effect sizes. Protocol heterogeneity across trials – in water temperature (8–20°C), immersion duration (5–20 min), body coverage, exercise type and HRV measurement timing – limited definitive pooling, but the directional consistency across 12 trials and multiple methodological implementations is a strong signal of a genuine and generalisable effect (Galvez‐Rodriguez et al., 2025).

Because the Galvez‐Rodriguez et al. (2025) systematic review is largely descriptive – aggregating the primary authors' conclusion statements with limited independent critical appraisal beyond risk‐of‐bias scoring – it is worth engaging the strongest primary evidence directly. The foundational controlled study by Buchheit et al. (2009) showed that brief immersion (5 min at ∼14°C) after high‐intensity exercise accelerated parasympathetic reactivation, with significantly faster recovery of vagally mediated HRV in the first 5–10 min post‐exercise than control. Stanley et al. (2012) extended this to the frequency domain, reporting that CWI hastened the return of high‐frequency (HF) spectral power and the normalisation of the LF/HF ratio toward resting values after endurance exercise, with carry‐over to next‐day submaximal performance. Across these trials the time‐domain index RMSSD and its frequency‐domain counterpart (HF power) move concordantly, as expected given that both quantify respiratory‐linked vagal modulation of the sinoatrial node, whereas the LF/HF ratio behaves less consistently owing to its recognised interpretive limitations. The principal caveats of this primary literature are small samples (typically *n* = 7–12), heterogeneous immersion and HRV‐recording protocols, and the impossibility of participant blinding, all of which temper the strength of the pooled signal even though its direction is reproducible (Buchheit et al., 2009; Stanley et al., 2012).

The clinical relevance of accelerated parasympathetic reactivation extends beyond subjective comfort. HRV‐guided training periodisation studies – in which training intensity on a given day is determined by the athlete's morning RMSSD relative to their personal rolling baseline – consistently demonstrate that HRV‐guided approaches produce equal or superior physiological adaptations compared with predefined load prescription, while reducing training stress markers and perceived fatigue (Kiviniemi et al., 2007). If CWI accelerates the return of RMSSD to personal‐baseline levels after a heavy training session, it may create additional windows for high‐quality training that would not exist under passive recovery – a genuine competitive performance advantage (Moore et al., 2022; Stanley et al., 2012). The caveat is that this perceptual–autonomic readiness may be partially decoupled from actual tissue repair state: an athlete with restored RMSSD may begin training before the satellite‐cell and mTORC1‐dependent repair and remodelling processes have reached the stage required for maximal quality contraction (Fyfe et al., 2019; Roberts et al., 2015). In a hypertrophy‐prioritised context, this temporal decoupling is itself an argument for limiting CWI, since it risks compressing the anabolic window of reduced‐load recovery that optimises molecular adaptation. Finally, the autonomic potency of cold exposure carries a safety corollary: in an observational study of recreational outdoor cold‐water swimmers, Faivre‐Rampant et al. (2024) documented post‐immersion QT‐interval lengthening of approximately 20 ms, exceeding 500 ms in 5 of 20 participants, underscoring that the same sympatho‐vagal perturbation that benefits recovery can transiently destabilise cardiac repolarisation in susceptible individuals; CWI should therefore be screened and supervised accordingly.

### 4.2 DOMS and perceived recovery

DOMS is mechanistically distinct from acute exercise pain: it peaks at 24–72 h post‐exercise, corresponds to the inflammatory phase of muscle fibre remodelling, and involves sensitisation of muscle mechanoreceptors and nociceptors by prostaglandins, bradykinin and substance P released from damaged fibres and infiltrating immune cells (Cheung et al., 2003; Peake et al., 2017). The 2024 network meta‐analysis by Chen et al. (2024) constitutes the most comprehensive ranking of recovery modalities for DOMS‐related outcomes. Pooling 57 RCTs and 1220 participants and using a Bayesian surface under the cumulative ranking curve (SUCRA) approach, the analysis ranked cryotherapy modalities first for relief of perceived muscle soreness (SUCRA 88.3%) and for neuromuscular performance recovery as assessed by countermovement jump height (SUCRA 83.7%). Contrast water therapy ranked highest for normalisation of circulating creatine kinase activity, a biochemical marker of sarcolemmal disruption. The Wang et al. (2022) network meta‐analysis of cold and heat therapies identified more nuanced temporal patterns: hot‐pack application ranked highest at 24 h post‐exercise, while contrast water therapy and CWI were comparably effective at 48 h, and a controlled‐gas whole‐body cryotherapy modality emerged as superior beyond 48 h. Consistent with these pooled findings, the recent randomised trial by Pereira et al. (2025) reported an absence of perceived pain at 48 h following ∼12°C, 10‐min CWI after a demanding CrossFit® ‘Murph’ workout, compared with massage, although the non‐blinded design and reliance on a single perceptual endpoint limit the strength of the inference.

The mechanism underlying the analgesic effect of cold modalities is multi‐factorial. Vasoconstriction‐mediated reduction of interstitial oedema reduces the mechanical activation of muscle mechanoreceptors (Schaser et al., 2007; Wilcock et al., 2006). Cooling slows sensory and motor nerve conduction in a temperature‐dependent manner, prolonging latency and contributing to cold‐induced analgesia (Lithfous et al., 2022; Machado et al., 2016). Suppression of COX‐2 and PGE2 production in cooled tissue attenuates the chemical sensitisation of peripheral nociceptors (Gleeson et al., 2011; Rousse et al., 2025). Together, these mechanisms produce an analgesic effect that is rapid in onset (within minutes of immersion), peaks at 24–48 h post‐exercise and is of moderate magnitude relative to pharmaceutical analgesia but with a superior safety profile for healthy athletic populations (Batista et al., 2023; Bleakley et al., 2012; Missau et al., 2018).

### 4.3 Modality differentiation: CWI versus contrast therapy versus whole‐body cryotherapy

A persistent source of confusion in the practitioner literature is the implicit treatment of CWI, contrast water therapy (CWT; alternating hot and cold immersion) and dry whole‐body cryotherapy chambers as pharmacologically interchangeable. The Chen et al. (2024) network meta‐analysis makes the disaggregation explicit: CWI and CWT differ in their ranking profiles across outcomes. CWT achieves higher mean SUCRA for creatine kinase normalisation (consistent with its promotion of hyperaemic flushing through hot–cold alternation, which may accelerate clearance of inflammatory mediators and metabolic by‐products from the interstitial space) but lower than cryotherapy for soreness and jump performance (Chen et al., 2024; Costello et al., 2016; Wang et al., 2022; Wu et al., 2026). Whole‐body cryotherapy chambers (−100°C to −140°C, 2–4 min) achieve substantially less intramuscular cooling than water immersion (approximately 1–2°C versus 3–5°C reduction in vastus lateralis temperature at depth) due to the low thermal capacity of cold air relative to cold water, and their anti‐anabolic potential is correspondingly lower (Wang et al., 2025). The dose–displacement implication is important: practitioners seeking analgesic benefit with minimal anabolic cost may achieve a reasonable trade‐off through CWT or abbreviated whole‐body cryotherapy, rather than the sustained whole‐body CWI that produces the deepest and most prolonged intramuscular cooling. The key findings regarding acute benefits for recovery and neuromuscular considerations are summarised in Table 1.

**TABLE 1:** Principal human evidence for the acute recovery benefits and post‐CWI neuromuscular considerations.

Study	Design	*n*	CWI protocol	Primary outcome	Key finding
Galvez‐Rodriguez et al. (2025)	Systematic review (12 RCTs)	Mixed	∼10–15°C, 10–15 min	RMSSD, HRV indices	All 12 studies: parasympathetic reactivation; 6 with *P *< 0.05 vs. passive; moderate‐to‐large ES
Chen et al. (2024)	Network meta‐analysis (57 RCTs, *n* = 1220)	1220	Various cryotherapy modalities	DOMS, CK, jump performance	CWI/cryotherapy SUCRA 88.3% for soreness; 83.7% for jump recovery; CWT best for CK
Wang et al. (2022)	Network meta‐analysis (cold/heat)	N/R	Various cold and heat modalities	Perceived DOMS at 24, 48, 72 h	Hot pack superior at 24 h; CWI/CWT comparable at 48 h; WBC superior >48 h
Cain et al. (2025)	Systematic review and meta‐analysis (11 RCTs)	Healthy adults	Various CWI protocols	Inflammation, stress, immunity, sleep	Significant ↑ inflammatory markers immediately and 1 h post‐CWI; relative ↓ at 12 h
Pereira et al. (2025)	RCT 2‐arm	30	∼12°C, 10 min post‐CrossFit® Murph	Perceived pain at 48 h	Absence of pain reports 48 h post‐CWI vs. massage; non‐blinded design
Heinke et al. (2024)	RCT 3‐arm	N/R	CWI vs. percussive massage vs. passive	CMJ height, neuromuscular performance	Acute CMJ decrement immediately after CWI; plausible motor nerve conduction slowing
Rousse et al. (2025)	Systematic review (combined therapies)	N/R	Cold, heat, hypoxia combinations	Muscle recovery biomarkers	CWI in combination with other stressors modulates inflammatory and remodelling cascades
Faivre‐Rampant et al. (2024)	Observational (*n* = 20)	20	Outdoor cold‐water swimming	QT interval, cardiac function	Post‐exposure QT lengthening ∼20 ms; 5/20 participants >500 ms; no overt cardiac events

Abbreviations: CK, creatine kinase; CMJ, countermovement jump; CWI, cold‐water immersion; CWT, contrast water therapy; DOMS, delayed onset muscle soreness; ES, effect size; HRV, heart rate variability; N/R, not reported; RCT, randomised controlled trial; RMSSD, root mean square of successive differences; SUCRA, surface under the cumulative ranking curve; WBC, whole‐body cryotherapy.

## 5 THE ADAPTATION SIDE OF THE PARADOX: MOLECULAR AND PHENOTYPIC EVIDENCE

### 5.1 mTORC1 as the central molecular target

mTORC1 is the master regulator of muscle protein synthesis in the post‐exercise anabolic cascade (Bodine, 2022). It integrates mechanical loading, transmitted through insulin receptor substrate 1 (IRS‐1)/phosphoinositide 3‐kinase (PI3K)/Akt (Glass, 2010) and the Piezo1 mechanosensitive channel (Greyvenstein et al., 2025); amino acid availability, sensed through Rag GTPases (Lama‐Sherpa et al., 2023); and endocrine signals including insulin‐like growth factor 1 (IGF‐1) via PI3K–Akt (Figueiredo & McCarthy, 2019; White, 2021). Activated mTORC1 phosphorylates p70S6K1 at Thr389 and 4E‐BP1 at Thr37/46, releasing the cap‐binding complex eukaryotic initiation factor 4F (eIF4F) and enabling cap‐dependent translation of contractile proteins including myosin heavy chain (Kimball & Jefferson, 2006). These targets set the rate at which the anabolic signal becomes new contractile protein and, ultimately, measurable fibre hypertrophy (Lim et al., 2022; Churchward‐Venne et al., 2012).

Cooling muscle by 2–5°C – the reduction achieved by 10–15 min of 10°C CWI – is sufficient to impair mTORC1 activation kinetics and downstream signalling (Roberts et al., 2015). The pivotal human in vivo evidence is the Fyfe et al. (2019) 7‐week resistance‐training RCT, in which 16 men performed 15 min of 10°C CWI or passive recovery after each of three weekly whole‐body sessions. Vastus lateralis biopsies at 1 and 48 h after the final session showed significantly lower p70S6K1 Thr389 phosphorylation in the CWI group (effect sizes −0.69 and −1.33), indicating sustained mTORC1 suppression. Protein‐degradation markers (FOX‐O1 and the E3 ubiquitin ligases MuRF‐1 and atrogin‐1) were elevated, and temperature‐dependent heat shock proteins (HSP27, HSP72) attenuated, consistent with a net shift toward catabolism (Fyfe et al., 2019).

### 5.2 Ribosomal biogenesis and translational capacity

At a level deeper than mTORC1 phosphorylation lies ribosomal biogenesis – the synthesis of new translational machinery needed to sustain the elevated protein synthesis of a growing muscle. Hypertrophy depends on chronic expansion of translational capacity (total ribosomal RNA per unit muscle), which sets the ceiling of synthetic output across weeks of training (Figueiredo & McCarthy, 2019; Lim et al., 2022). Rantala & Chaillou (2019) provide the most direct data: human primary myotubes held at 32°C versus 37°C for 48 h showed reduced protein synthesis, significantly lower 45S pre‐ribosomal RNA (a marker of rDNA transcription rate) and reduced MYC content; glutamine‐induced hypertrophic responses were also blunted, implicating impaired nutrient‐sensing alongside mTORC1 suppression (Rantala & Chaillou, 2019; Churchward‐Venne et al., 2012). These in vitro data await human in vivo replication and must be extrapolated with caution.

### 5.3 Satellite cell signalling and myonuclear domain expansion

Satellite cells – muscle‐resident progenitors occupying the sub‐basal‐lamina space of myofibres – supply the new myonuclei that support growing fibres. Mechanically damaged fibres release hepatocyte growth factor (HGF), fibroblast growth factor and NO, driving quiescent Pax7^+^ satellite cells into proliferation, differentiation and fusion into the host fibre (Chargé & Rudnicki, 2004). This expands the myonuclear domain and becomes rate‐limiting for hypertrophy beyond early training. The inflammatory cascade – IL‐6, HGF and IGF‐1 signalling, all modulated by CWI – is an essential upstream activator of satellite‐cell mobilisation (Chargé & Rudnicki, 2004; Serrano et al., 2008).

Although direct satellite‐cell counts across a full post‐CWI training cycle are not yet published, indirect evidence from Fyfe et al. (2019) is consistent with attenuated myonuclear addition: type II fibre cross‐sectional area increased significantly less with CWI than passive recovery after 7 weeks of matched training (effect size −1.37), despite comparable workload. Preserved stimulus with reduced hypertrophy is the canonical signature of constrained myonuclear‐domain expansion (Lim et al., 2022; Chargé & Rudnicki, 2004). Satellite‐cell activation is itself temperature‐sensitive, depending on enzyme kinetics and on the very prostaglandin and cytokine signals attenuated by CWI‐mediated cooling (Roberts et al., 2015).

### 5.4 Longitudinal strength and hypertrophy outcomes: The meta‐analytic picture

The translation from molecular signalling to longitudinal phenotype is the question that most directly concerns applied practitioners. Grgic's (2023) meta‐analysis of 10 trials and 11 comparisons in primarily male resistance‐trained populations remains the most comprehensive synthesis of longitudinal CWI effects on strength and hypertrophy outcomes. Post‐exercise CWI significantly attenuated muscular strength gains relative to passive recovery (effect size −0.23; 95% CI: −0.45 to −0.01; *P* = 0.041). The effect was concentrated in and substantially larger for trials using limb‐specific CWI (effect size −0.31; 95% CI: −0.61 to −0.01; *P* = 0.041), whereas whole‐body CWI produced a non‐significant trend. This was complemented by the Piñero et al. (2024) systematic review and meta‐analysis specifically focusing on hypertrophy: pooling eight studies, they found strong evidence for greater relative hypertrophic adaptations with resistance training (RT) alone compared to CWI + RT (effect size = −0.22; 95% CI: −0.47 to 0.04), concluding that post‐exercise CWI may attenuate hypertrophic changes (Piñero et al., 2024). This specificity pattern – limb CWI more anti‐anabolic than whole‐body CWI – is consistent with a model in which the relevant parameter is the local muscle‐temperature reduction achieved in the trained muscles, rather than systemic cold exposure or generalised autonomic/inflammatory effects. The Fyfe et al. (2019) trial contributed the most mechanistically detailed data point: whole‐body CWI did not significantly reduce one‐repetition maximum leg‐press strength over 7 weeks, but substantially reduced type II fibre cross‐sectional area (CSA) – a dissociation consistent with the multi‐determined nature of maximal strength (which includes neural drive, motor unit synchronisation and rate of force development components independent of fibre CSA) versus hypertrophy (which is primarily determined by cumulative net protein balance).

### 5.5 Acute neuromuscular performance impairment

CWI can acutely impair neuromuscular performance in the 1–2 h window after exposure, a concern for same‐day multi‐session or tournament contexts. The RCT by Heinke et al. (2024) compared CWI, percussive massage and passive rest after exhausting eccentric exercise and reported acute decrements in countermovement‐jump (CMJ) height with CWI relative to percussive massage (Heinke et al., 2024). The plausible mechanism is reduced motor‐nerve conduction velocity (∼1.5–2.0 m s^−^^1^ per °C of nerve cooling) and slowed cross‐bridge kinetics (Bleakley et al., 2012; Lithfous et al., 2022). CWI applied to lower‐limb musculature immediately before explosive tasks (sprinting, jumping, change of direction) may therefore impair rather than enhance performance in the first 60–90 min (Parouty et al., 2010; Richards et al., 2025).

### 5.6 The NSAID parallel: A conceptual framework

The CWI picture maps onto the established literature on non‐steroidal anti‐inflammatory drugs (NSAIDs) and resistance‐training adaptation, a useful practitioner analogy. Both provide acute analgesic and anti‐inflammatory benefit by suppressing the prostaglandin and cytokine cascade, and both, applied across the post‐exercise anabolic window, attenuate satellite‐cell activation and protein synthesis – reducing hypertrophic adaptation more consistently than maximal strength while remaining context‐specific rather than uniformly harmful (Fyfe et al., 2019; Gleeson et al., 2011; Trappe et al., 2011; Grgic, 2023; Piñero et al., 2024).

## 6 ENDURANCE ADAPTATIONS: WHERE THE PARADOX IS MORE SUBTLE

The endurance literature suggests a qualitatively different and more favourable picture for CWI. This difference is consistent with the mechanistic divergence between the two adaptive programmes. Anabolic protein‐synthetic pathways are driven by mechanical loading, are mTORC1‐dependent and are temperature‐sensitive; oxidative‐mitochondrial pathways are driven by metabolic perturbation and by calcium/AMP‐activated protein kinase (AMPK) signalling, and they appear to be partially cold‐activated rather than cold‐suppressed (Ihsan et al., 2016; Puigserver & Spiegelman, 2003).

The direct evidence is anchored by the Ihsan et al. (2020) within‐subject trial, in which nine men performed 3‐weekly endurance sessions for 4 weeks with one leg receiving 15 min of 10°C CWI after each session and the contralateral leg passive recovery. The CWI‐treated leg exhibited significantly higher microvascular reperfusion (near‐infrared spectroscopy and contrast‐enhanced ultrasound), consistent with enhanced vascular remodelling exceeding the untreated leg (Ihsan et al., 2020). This contradicts a simplistic ‘CWI suppresses adaptation’ narrative. The most plausible account is hormetic: repeated vasoconstriction during CWI followed by reactive hyperaemia forms a vascular training stimulus that may amplify angiogenesis, possibly through vascular endothelial growth factor and NO release during the post‐immersion hyperaemic phase (Ihsan et al., 2020; Rowell, 1974).

At the molecular level, cold exposure activates PGC‐1α – the master regulator of mitochondrial biogenesis – through β3‐adrenergic signalling, AMPK and sirtuin 1 (SIRT1) in animal models (Cannon & Nedergaard, 2004; Puigserver & Spiegelman, 2003). If cold engages signals overlapping with endurance exercise through a parallel pathway, it may enhance mitochondrial adaptation after endurance bouts; this is directionally supported by Ihsan et al. (2020) but not yet confirmed by human biopsy studies (Ihsan et al., 2020; Hornberger, 2011).

An important boundary condition applies. This favourable picture concerns low‐to‐moderate‐intensity continuous endurance training, where mitochondrial biogenesis and capillary density dominate and are driven by AMPK, Ca^2+^/calmodulin‐dependent protein kinase II (CAMKII) and SIRT1 signalling that may be cold‐compatible or cold‐enhanced. In high‐intensity interval training (HIIT), muscle damage, inflammatory signalling and satellite‐cell mobilisation play a larger role in the secondary remodelling that contributes to aerobic gains (Konopka et al., 2011; Lira et al., 2017). CWI after HIIT may suppress this component as it does in resistance training, introducing a partial adaptation cost even in endurance contexts (Rousse et al., 2025; Richards et al., 2025). The endurance literature is therefore not monolithic, and the decision framework stratifies endurance–CWI decisions by training‐intensity domain.

A further consideration for endurance athletes concerns the thermoregulatory application of CWI – its use primarily to manage heat strain in warm‐environment training and competition, facilitate sleep onset on competition nights and manage perceived recovery during multi‐day events (Cain et al., 2025; Ihsan et al., 2016; Versey et al., 2013). This thermoregulatory deployment falls outside the recovery–adaptation paradox in its strict form because the primary physiological target is heat dissipation rather than anabolic signalling. The longitudinal adaptation cost of CWI in a thermoregulatory context – where CWI is used selectively and in response to heat stress rather than systematically after every training session – is presumed to be small relative to the performance gains from mitigating heat fatigue, but this presumption has not been formally tested in dedicated longitudinal trials.

## 7 CONCURRENT TRAINING, SEX DIFFERENCES AND TRAINING‐STATUS MODULATION

### 7.1 Concurrent training

Concurrent training – the simultaneous pursuit of strength/hypertrophy and endurance adaptations – is the modal structure for team‐sport athletes, military personnel and recreational trainees, and the most complex CWI application because its two adaptive targets (mTORC1‐driven hypertrophy and AMPK/PGC‐1α‐driven mitochondrial biogenesis) have opposing sensitivities to post‐exercise cooling (Fyfe et al., 2019; Ihsan et al., 2016; Puigserver & Spiegelman, 2003). Prescription therefore requires explicit goal prioritisation rather than a single blanket recommendation.

When hypertrophy and strength are primary adaptive goals in a concurrent training programme, CWI should be withheld after resistance‐training sessions and may be used selectively after endurance sessions. This goal‐specific deployment exploits the differential mechanistic sensitivity of anabolic versus oxidative pathways: the anabolic pathway (mTORC1/p70S6K1) is substantially more temperature‐sensitive than the mitochondrial biogenesis pathway (AMPK/PGC‐1α) (Fyfe et al., 2019; Roberts et al., 2015). Withholding CWI after resistance sessions preserves the full mTORC1 signalling window, while allowing CWI after endurance sessions may be neutral‐to‐beneficial for microvascular adaptation and acceptable for mitochondrial targets (Ihsan et al., 2020; Rousse et al., 2025). When endurance capacity is primary in a concurrent programme – as in team‐sport pre‐season blocks or military operational fitness training – CWI may be used after endurance sessions with less concern for adaptive cost, though the caveat about HIIT‐induced secondary remodelling applies (Elias et al., 2013; Malta et al., 2021; Richards et al., 2025).

### 7.2 Sex differences in CWI response

The CWI literature is dominated by young men, limiting generalisability to women, for whom several non‐speculative physiological differences exist (Haizlip et al., 2015; O'Bryan et al., 2022). Women show lower baseline muscle protein synthesis under equivalent loading, reflecting differences in anabolic hormones, lean mass and myosin heavy‐chain isoform distribution that may alter both the mTORC1 response and its CWI‐mediated attenuation (Churchward‐Venne et al., 2012; Witard et al., 2014). Thermoregulatory vasoconstriction is more extensive in women owing to lower lean mass and surface‐area‐to‐mass ratio, potentially amplifying CWI's peripheral vascular and cooling effects (Burse, 1979; Castellani & Young, 2016; Hohenauer et al., 2022; Straat et al., 2022). Menstrual cycle phase also modulates inflammatory and immune responses, adding an uncharacterised interaction with the CWI inflammatory axis (Notbohm et al., 2023). The framework should be applied to women as a reasonable default, aware that magnitudes of benefit and cost may differ systematically from male‐derived estimates.

### 7.3 Training status and adaptation ceiling

Training status is a second modulator. In untrained individuals the mTORC1 response to a given resistance stimulus is substantially larger (two‐ to five‐fold in some biopsy studies), as are GLUT4 and mitochondrial responses to endurance training (Dreyer et al., 2010; Holloszy, 2011; Dungan, 2017; Ato et al., 2019). The anti‐anabolic cost of CWI – attenuating part of this signal – may therefore exert a proportionally smaller phenotypic effect in novices with abundant adaptive reserve than in well‐trained athletes near their adaptation ceiling (Figueiredo & McCarthy, 2019; Lim et al., 2022). Piñero et al. (2024) found no significant moderation of the CWI hypertrophy effect by training status (β = −0.10; 95% CI: −0.65 to 0.43; *P* = 0.653), though power was limited. Conversely, in elite athletes maximising hypertrophy late in a mesocycle, the same mTORC1 attenuation may represent a meaningful fraction of residual adaptive capacity; Grgic (2023) reported larger, more consistent effects in longer interventions (>8 weeks) and more trained populations, consistent with a ceiling‐effect model.

## 8 INTEGRATIVE SYNTHESIS: A PROTOCOL‐BY‐GOAL DECISION FRAMEWORK

The mechanistic and longitudinal evidence reviewed in sections 3.2, 4.2, 5.2, 7.1, 7.2 supports a decision framework in which the appropriate use of CWI depends on three interacting variables: (i) the primary adaptive goal of the training cycle or session, (ii) the protocol parameters of CWI exposure (temperature, duration, body coverage, timing), and (iii) the temporal placement of CWI relative to the anabolic training stimulus. Table 2 operationalises this framework across seven training contexts. Four principles, summarised in a single paragraph below, integrate the evidence.

**TABLE 2:** Protocol‐by‐goal decision framework for post‐exercise cold‐water immersion in trained adult populations.

Training context/goal	CWI recommendation	Suggested parameters	Mechanistic rationale
Acute inter‐session recovery (multi‐session day, tournament)	Recommended	10–15°C; 10–15 min; whole/partial body; within 30 min post‐session	Maximises parasympathetic reactivation and analgesic effect; anabolic cost minimal over single day
Primary hypertrophy mesocycle	Avoid (systematic use)	Omit or delay >8 h; limit to occasional use if essential	Repeated mTORC1 suppression + reduced ribosomal biogenesis + attenuated type II CSA gains
Strength‐focused block (off‐season)	Limit to non‐training days	Use only on rest days or >8 h after RT session	Limb‐specific cooling immediately post‐RT is the primary mediator of anti‐anabolic effect
In‐season competition (strength‐hypertrophy)	Selective use on competition days	Apply CWI post‐competition only; avoid post‐RT in developmental sessions	Decouples analgesic benefit from anabolic cost by restricting CWI to non‐developmental windows
Endurance base/specific phase	Cautious use	10–15°C; 10–15 min; post key sessions; preserve 1–2 non‐CWI sessions/week	Microvascular and mitochondrial adaptations appear preserved or enhanced; longitudinal evidence limited
Concurrent training (hypertrophy primary)	Withhold post‐RT sessions	Omit CWI after resistance sessions; permissible after endurance bouts	Differential mechanistic sensitivity of anabolic vs. oxidative‐mitochondrial pathways
Concurrent training (endurance primary)	Permissible post‐endurance	Standard parameters after endurance; avoid immediately post‐RT	Endurance pathways (AMPK, PGC‐1α) less affected than mTORC1 anabolic pathway

Abbreviations: AMPK, AMP‐activated protein kinase; CWI, cold‐water immersion; ES, effect size; HIIT, high‐intensity interval training; MPS, muscle protein synthesis; mTORC1, mechanistic target of rapamycin complex 1; PGC‐1α, peroxisome proliferator‐activated receptor γ coactivator 1‐α; RT, resistance training.

First, the paradox is not a contradiction but a predictable mechanistic divergence: the acute neural–vascular benefit (first 1–4 h) and the chronic anti‐anabolic cost (accumulating over weeks) act on different time scales and coexist (Cain et al., 2025; Fyfe et al., 2019; Galvez‐Rodriguez et al., 2025; Piñero et al., 2024; Roberts et al., 2015). Second, the anti‐anabolic cost is dose‐dependent – greatest for limb‐specific CWI applied immediately after resistance training, smaller for whole‐body or delayed exposure, and smallest for brief, warmer or partial‐body protocols (Grgic, 2023; Roberts et al., 2015). Third, the cost is pathway‐specific: anabolic, mTORC1‐dependent targets are highly cold‐sensitive, whereas mitochondrial, microvascular and neural targets are largely spared or even enhanced (Cannon & Nedergaard, 2004; Ihsan et al., 2020; Puigserver & Spiegelman, 2003; Rantala & Chaillou, 2019). Fourth, the conventional 10–15°C, 10–15 min protocol was optimised for perceived recovery rather than for the analgesia‐versus‐adaptation trade‐off, so warmer water (15–18°C), shorter duration (5–8 min) or shallower immersion are reasonable strategies to preserve hypertrophy while retaining some analgesic benefit (Grgic, 2023; Machado et al., 2016; Vieira et al., 2016).

The framework translates these principles into context‐specific guidance, which Table 2 summarises. For acute inter‐session recovery (multi‐session days, tournaments, congested fixtures), CWI is recommended at 10–15°C for 10–15 min within ∼30 min of the session, because the autonomic and analgesic benefits dominate and a single day's anabolic cost is trivial. During a primary hypertrophy mesocycle, systematic post‐resistance CWI should be avoided or delayed beyond ∼8 h, because repeated mTORC1 suppression accumulates into measurable losses of type II fibre cross‐sectional area. In strength‐focused blocks the cost is smaller, because maximal strength is multiply determined, so CWI can be restricted to rest days or to sessions well separated from resistance training. In endurance phases, CWI may be used after key sessions while preserving one to two CWI‐free sessions per week, given that microvascular and mitochondrial adaptations appear preserved or enhanced. In concurrent training, the decision follows the prioritised goal: withhold CWI after resistance sessions when hypertrophy is primary but allow it after endurance sessions.

The framework rests on a final observation: the acute markers practitioners use to judge CWI (RMSSD, perceived soreness, perceived freshness) do not track the chronic adaptive cost (fibre CSA, muscle protein synthesis rates, ribosomal biogenesis) (Fyfe et al., 2019; Piñero et al., 2024; Roberts et al., 2015). A practitioner who judges CWI by next‐session readiness will miss the mesocycle‐level cost until it manifests as a hypertrophy plateau relative to a matched non‐CWI group. Monitoring body composition (dual‐energy X‐ray absorptiometry or ultrasound‐derived muscle thickness) across the mesocycle is the most practical way to integrate this evidence into applied monitoring (Lim et al., 2022).

## 9 RESEARCH GAPS AND FUTURE DIRECTIONS

Several priorities would most rapidly close the evidence gaps. First, sex‐stratified longitudinal RCTs: the available trials are dominated by young men, yet sex differences in baseline protein synthesis, menstrual‐cycle‐modulated inflammation, cold‐shock vasoconstriction and anabolic hormones may alter both the anti‐anabolic cost and the analgesic benefit in women, so dedicated RCTs in resistance‐trained females – with menstrual‐cycle phase controlled – are needed (Notbohm et al., 2023). Second, older and sarcopenic populations: anabolic resistance defines ageing muscle, so repeated post‐exercise CWI could impose a proportionally larger, clinically consequential anti‐anabolic cost, yet no RCT has tested CWI and resistance‐training adaptation in adults over 60 (Anagnostou et al., 2025; Witard et al., 2014). Third, dose–response optimisation: the surface relating water temperature, duration, depth and timing to the analgesia‐versus‐adaptation ratio has not been mapped, and one‐parameter‐at‐a‐time trials with both molecular and longitudinal outcomes would enable individualised prescription (Machado et al., 2016; Vieira et al., 2016). Fourth, endurance‐specific performance endpoints: the endurance evidence rests on indirect microvascular biomarkers without long‐term ${\dot{V}}_{O_{2}\max}$ or performance outcomes, requiring 10–16‐week trials to confirm whether microvascular enhancement translates into performance and to characterise HIIT‐related costs (Ihsan et al., 2020; Ihsan et al., 2016). Fifth, mechanistic individual‐response characterisation: population means conceal heterogeneity in cold‐shock responsiveness, sympathetic reactivity, BAT activity, mTORC1 responsiveness and satellite‐cell pool, and phenotype‐stratified studies would advance personalised prescription (Cannon & Nedergaard, 2004; Figueiredo & McCarthy, 2019; Lim et al., 2022). Finally, concurrent‐training and real‐world contexts remain under‐studied in elite athletes under ecologically realistic loads; season‐long studies integrating objective training‐load, recovery and performance monitoring would improve applicability (Elias et al., 2013; Moore et al., 2022; Rousse et al., 2025; Stanley et al., 2012).

## 10 CONCLUSIONS

CWI is neither uniformly helpful nor uniformly harmful. It is a powerful, dose‐dependent cold stressor whose effect on training adaptation depends on the goal, the protocol and the timing relative to training. The central paradox of this review is not a contradiction: through the same molecular pathways, CWI speeds the autonomic and vascular recovery that matters most in the first 24–72 h after exercise, while blunting the mTORC1‐driven muscle‐building processes that accumulate over weeks of resistance training.

Both sides of the paradox are now supported. On the acute side, the evidence is unusually consistent: CWI reliably speeds parasympathetic reactivation, reduces perceived soreness and swelling at 24–72 h, and shifts the inflammatory time course in a direction favourable for next‐session readiness. On the adaptation side, the molecular evidence is specific and the longitudinal evidence, although still limited in scale, is internally consistent: repeated post‐resistance CWI lowers p70S6K1 phosphorylation and ribosomal‐biogenesis markers, reduces type II fibre hypertrophy, and produces a small but significant attenuation of strength gains (effect size ≈ −0.23). This cost is greatest with limb‐specific CWI applied immediately after resistance training – the configuration that cools the trained muscle most deeply during peak mTORC1 activation.

The protocol‐by‐goal framework presented in Table 2 operationalises these conclusions into actionable decisions across seven clinically relevant training contexts. It rests on the principle that the appropriate use of CWI should be determined by adaptive goal, not by habit, social norm, or the acute perceptual attractiveness of the modality. Where acute inter‐session recovery is the dominant requirement, CWI should be used at its conventional, well‐validated dose; where hypertrophic adaptation is the dominant requirement, it should be withheld, delayed, or attenuated in dose; and where both goals are pursued concurrently, the framework directs CWI toward the endurance or competition‐day component of training and away from the resistance‐training component.

The priorities that will most rapidly close the remaining gaps are longitudinal RCTs in female and older‐adult populations: systematic dose–response trials varying CWI parameters against molecular and phenotypic outcomes; endurance‐performance trials long enough to detect ${\dot{V}}_{O_{2}\max}$ change; and mechanistic stratification studies linking individual biological phenotypes to differential CWI response. Until those gaps are filled, CWI should be treated as what the evidence demonstrates – an active intervention with a definable cost–benefit profile, to be prescribed by goal and protocol rather than adopted as a reflexive post‐exercise default.

## AUTHOR CONTRIBUTIONS

Jose Francisco Tornero‐Aguilera: conceptualisation, data curation, formal analysis, investigation, methodology, project administration, validation, writing – original draft, writing – review & editing. Jose Lozano‐Meca: data curation, formal analysis, investigation, software, validation, writing – review & editing. Miguel López‐Moreno: data curation, investigation, software, writing – original draft. Edgar Simón Sancho‐Haro: data curation, investigation, visualisation, writing – original draft. Mario Muñoz‐López: formal analysis, investigation, software, validation, writing – review & editing. Eneko Baz‐Valle: formal analysis, investigation, software, validation, visualisation, writing – review & editing. José Francisco López‐Gil: investigation, methodology, validation, visualisation, writing – review & editing. Rodrigo Yáñez‐Sepúlveda: formal analysis, investigation, methodology, validation, visualisation, writing – review & editing. Vicente Javier Clemente‐Suárez: conceptualisation, funding acquisition, methodology, resources, supervision, validation, writing – review & editing. All authors have read and approved the final version of the manuscript. All authors have read and approved the final version of this manuscript and agree to be accountable for all aspects of the work in ensuring that questions related to the accuracy or integrity of any part of the work are appropriately investigated and resolved. All persons designated as authors qualify for authorship, and all those who qualify for authorship are listed.

## CONFLICT OF INTEREST

None declared.

## FUNDING INFORMATION

None.

## GENERATIVE AI STATEMENT

The authors used generative AI tools only for limited copy‐editing support (grammar, syntax and style refinement). No content, interpretation, mechanistic synthesis or reference generation was produced by AI tools. The authors take full responsibility for the scientific content of the manuscript.

## Data Availability

Data sharing is not applicable to this article, as no datasets were generated or analysed during this narrative review. All cited sources are publicly indexed in PubMed and accessible via their digital object identifiers.
